# ‘Social media comes with good and bad sides, doesn’t it?’ A balancing act of the benefits and risks of social media use by young adults with long-term conditions

**DOI:** 10.1177/13634593211023130

**Published:** 2021-06-03

**Authors:** Ceri Wilson, Jennifer Stock

**Affiliations:** Anglia Ruskin University, UK; King’s College London, UK; Bethlem Royal Hospital, UK

**Keywords:** chronic illness, long-term conditions, online communities, social media, young adulthood

## Abstract

Young adults are frequent users of social media, but the help and hindrance of social media for living well with long-term conditions (LTCs) in young adulthood is little-researched. The aim of this paper was to explore the experiences of social media use amongst young adults with LTCs. Interviews with 15 young adults with LTCs explored their experiences of using social media more broadly and in relation to online health communities. Social media came with both ‘good and bad sides’ which required a balancing act to manage (overarching theme), as reflected in the following subthemes: (1) Relationships: reducing social isolation versus need for face-to-face contact; (2) Comparisons: normalising versus negative (upward) comparisons; (3) Community: fitting in versus feeling left out; (4) Emotions: inspiring versus distress contagion; and (5) Knowledge: exchanging useful information versus fear of decline. The findings highlight the importance of young adults’ self-reflection/awareness of social media’s impact on their wellbeing, identifying when limited or increased use may be preferable. Whilst there is a ‘good’ to social media such as increased feelings of belonging and connection, this should not be the sole focus of future self-management interventions; as its use also contributes to feelings of distress, fear and not fitting in, and participants desire face-to-face contact.

## Introduction

Long-term conditions (LTC), or chronic illnesses, are medical conditions such as diabetes, epilepsy and Crohn’s Disease, that can be controlled but not cured and require management of complex symptoms and treatments (Department of Health [DH], 2012). According to Erikson’s (1963, 1968) stages of psychosocial development young adulthood comprises ages 18–40, and whilst the likelihood of having LTCs increases with age there is a significant proportion of young adults living with these conditions (15% of adults aged 20–29 and 20% aged 30–39: DH, 2012). However, the experiences and management strategies of young adults with LTCs are under-researched compared to other age groups.

Social networking sites (SNSs) such as Facebook and Instagram, have grown in popularity exponentially in recent years; particularly amongst young adults. Facebook alone has 2.23 billion monthly active users (Facebook Newsroom, 2018), 54% of whom are aged 18–34 (Statista, 2018). In America 88% of 18–29-year-olds use some form of social media, with 80% using Facebook and 71% using Instagram (Pew Research Center, 2018), and in the UK 91% of 16–24-year-olds use social media (Office for National Statistics, 2016). Of further note, time spent on SNSs is reported to have increased by 44% worldwide since the onset of the Covid-19 pandemic (Statista, 2020). While social media presents opportunities for innovation, self-expression and creativity; public concern about its impact on young people’s wellbeing has been well-documented (e.g. Royal Society of Public Health [RSPH], 2019).

A number of online groups aimed at supporting individuals with LTCs have arisen on SNSs. These online health communities are increasingly being used by individuals to support their self-management. Conrad et al. (2016) postulate how the internet has changed illness experiences through the development of illness subcultures and collective illness identities through online health communities. The development of such subcultures and collective identities may foster feelings of belonging, which is considered a deep emotional need and a key determinant of health and wellbeing (Bernat and Resnick, 2009; Yuval-Davis, 2004). This notion is supported by a growing body of research highlighting social and emotional benefits of online health communities; however, negative impacts are also being reported.

The following benefits of online health communities for individuals with specific LTCs of various ages have been reported: the development of reciprocally supportive relationships; reduced isolation/loneliness; reduced depression/increased emotional support; useful experiential knowledge-sharing; and feeling ‘normal’ (e.g. Allen et al., 2016; Caron and Light, 2015; Kingod et al., 2017; Kohut et al., 2017; Meade et al., 2018; Patel et al., 2015; Shavazi et al., 2016; Shoebotham and Coulson, 2016; Sinha et al., 2018; Smedley et al., 2015).

Reported negative impacts include: sharing of inaccurate information; negative aspects of conditions becoming visible; users feeling dissatisfied with social support remaining online; worries around trusting people online and knowing how much to share; and being upset by negative experiences shared in the community, sometimes referred to as ‘distress contagion’ (e.g. Allen et al., 2016; Caron and Light, 2015; Kingod et al., 2017; Kohut et al., 2017; Patel et al., 2015; Shoebotham and Coulson, 2016).

One study of relevance has been conducted exclusively with young adults. Fergie et al. (2015, 2016) conducted interviews with 40 young adults (aged 18–30) with diabetes or mental health problems exploring their online use. Aligning with earlier mentioned findings, the young adult participants in Fergie et al.’s (2015, 2016) research talked about the benefits of condition-specific Facebook pages for: seeking others with similar health conditions, sharing research, feeling less isolated, having experiences validated, provision of emotional support, and useful sharing of experiential information. However, as found elsewhere, the participants also noted that these communities can be depressing and cause ‘distress contagion’. Some participants also described their social media use more generally, reporting being wary of being seen as a ‘moaner’ if they posted regularly about their health.

To date, little research in this field has been conducted with young adults specifically, and the majority of existing research exploring social media use amongst people with LTCs has focused on the use of online health communities, with little attention to the impact of broader social media use. We know, however, that social media use in healthy young adults can have both positive impacts such as increased social support, reduced social anxiety and opportunity for self-expression (Ellison et al., 2007; RSPH, 2019); and negative impacts such as anxiety, depression, and envy when making social comparisons leading to lower self-esteem and wellbeing (Gothenburg Research Institute, 2012; RSPH, 2019). The latter impact can be further unpicked through looking to social comparison theory which posits that people seek to compare themselves to others to determine their own levels of abilities and success (Festinger, 1954), and the resulting comparison impacts on self-image and wellbeing. Downward social comparisons involve comparing to ‘inferior’ others, and lateral comparison involves comparison to similar others. Upward social comparison, on the other hand, involves comparison with ‘superior’ others, and leads to the individual finding a discrepancy between the self and the comparison standard. This is also sometimes referred to as negative social comparisons (e.g. de Vries and Kühne, 2015). These negative comparisons are thought to motivate the individual to change to be more like the comparison standard and therefore improve the self (Higgins, 1987; Wood, 1989). However, self-esteem is harmed when individuals believe that they cannot overcome their inferiority (Smith, 2000; Wills, 1981). It has been argued that the likelihood of engaging in social comparison is more intense in the social media sphere than in everyday life, as individuals are exposed to many comparison targets and a plethora of information about the desirable aspects of each comparison target’s life (e.g. Appel et al., 2016; Charoensukmongkol, 2018). It is widely cited that social media content usually comprises socially desirable information that people have posted to present themselves positively, and when others are exposed to this, they can experience feelings of envy due to upward social comparison to these seemingly advantaged others (e.g. Appel et al., 2016; Chou and Edge, 2012). Evidence suggests that amongst young adults in particular, more intense use of SNSs is related to more frequent negative social comparisons (e.g. de Vries and Kühne, 2015; Lee, 2014) and in the wider population findings suggest that social comparison mediates the impact of social media use on depression, envy and low self-esteem (e.g. Appel et al., 2016; Steers et al., 2014).

It seems likely that the reported positive and negative impacts of using SNSs for healthy young adults are likely to be more pronounced for young adults with LTCs. For example, the importance of social support potentially offered through SNSs could be more valued and important for young adults with LTCs; in light of their increased feelings of social isolation and disadvantage (e.g. Buchanan et al., 2010; Reddihough et al., 2013; Sattoe et al., 2014). Likewise, the negative consequences of upward social comparison through these sites could have a greater impact on young adults with LTCs as they report not meeting societal expectations and goals for their age, which is a cause of worry and dissatisfaction (e.g. Tunnicliffe et al., 2016; Wilson and Stock, 2019). Therefore, the impact of social media on young adults with LTCs requires further investigation.

The aim of this paper was to explore the experiences of social media use amongst young adults with LTCs. The data were collected pre-pandemic, but due to increased online social interactions since the onset of the pandemic, the need for research into the impact of social media is more pertinent that ever. The data reported form part of a wider study which explored the broader experience of living with LTCs in young adulthood and what helps and hinders living well with LTCs. The current article focuses specifically on the research question: what help and hindrance does social media play in living well with LTCs in young adulthood? The uniqueness of this paper is that it explores social media use for young adults specifically (with a range of LTCs) and explores not only the use of online health communities, but also the broader use of social media.

## Methods

### Design

In depth semi-structured one-to-one interviews were conducted with young adults with various LTCs to understand their experience of using social media, in the context of limited existing research with this population. Thematic analysis (Braun and Clarke, 2006) was used to inductively identify themes about the role social media can play in helping and/or hindering young adults to live well with their LTCs.

### Participants

To be eligible to participate individuals had to be aged 18–40, speak English, and currently living with one or more LTCs for a minimum of 6 months. Individuals were not eligible to participate if they had extensive cognitive impairment affecting their capacity to independently care for themselves. Participants comprised 10 females and five males, aged 19–39. The majority lived with more than one LTC (see Table 1).

**Table 1.** Participant demographics.

Pseudonym	Gender	Age	Employment status	Long-term condition/s	Time since diagnosis
‘Ruth’	Female	30	Part-time employed	Chronic fatigue syndrome	6 years
				Asthma	2 years
				Fibromyalgia	<6 months
‘Rebecca’	Female	24	Part-time employed	Microprolactinoma	4 years
				Chronic fatigue	4 years
				Connective tissue autoimmune disease	<6 months
‘John’	Male	39	Part-time self-employed	Post-traumatic stress disorder	4 years
				Eating disorder	Not specified (‘for years’)
‘Anna’	Female	25	Full-time employed	Crohn’s disease	10 years
‘Elizabeth’	Female	29	Part-time employed	Chronic fatigue syndrome	2 years
				Fibromyalgia	2 years
				Irritable bowel syndrome	2 years
‘Samantha’	Female	27	Full-time self-employed	Lupus nephritis	4 years
				Systemic lupus	4 years
				Chronic migraines	‘Since childhood’
				Depression	6 years
‘Ella’	Female	29	Full-time employed	Thyroid disease (and removal)	18 years
				Chronic fatigue	18 years
‘James’	Male	32	Full-time postgraduate student	Irritable bowel syndrome (IBS-D)	10 years
				Social anxiety disorder	10 years
‘Matt’	Male	26	Full-time undergraduate student	Chronic fatigue syndrome (later changed to below diagnosis)	14 years
				Benign brain cyst	7 years
‘Chloe’	Female	31	Full-time employed	Chronic fatigue syndrome	12 years
‘Zoe’	Female	19	Full-time undergraduate student	Type one diabetes	12 years
‘Luke’	Male	32	Part-time employed	Neuropathy	29 years
‘Alice’	Female	20	Full-time undergraduate student	Asthma	4 years
				Depression	4 years
				Anxiety	4 years
‘Tom’	Male	31	Full-time employed	Multiple sclerosis (relapse and remitting)	7 years
				Chronic fatigue	7 years
				Depression	7 years
‘Emily’	Female	26	Part-time employed	Grave’s disease (and thyroid removal)	4 years
				Depression	6 years
				Anxiety	6 years
		Mean: 28			Mean: 9.6 years

Some participants had a primary diagnosis of chronic fatigue syndrome while others experienced chronic fatigue as a result of their primary diagnosis. There were also a number of participants who reported experiencing clinically diagnosed depression and/or anxiety (as indicated in table) but a further two reported undiagnosed/self-reported anxiety and/or depression.

### Procedure

Ethical approval was obtained from the Anglia Ruskin University Faculty Research Ethics Panel. A purposive sampling approach was used to recruit participants. We recruited from local counties in the East of England through: UK-based charities for LTCs; social media; and through the Anglia Ruskin University website. Interviews were conducted by the first author and took place either at one of the Anglia Ruskin University campuses or in a community venue at a more convenient location for the participant. Participants provided written informed consent prior to taking part. The interview, lasting approximately 1 hour, explored the experiences of living with the condition/s, impact of the condition/s on different aspects of life, and what helps and hinders living well with the condition/s. A specific question was asked related to social media: ‘Do you feel that social media has any role to play in helping you to live well with your LTC/s’? Some participants also talked about social media in their answers to other questions about the impact of the condition/s and their self-management strategies. As interviews involved discussion of sensitive topics, if and when participants became upset the interviewer offered to stop the interview, offered a break and/or skipping a question. Interviews were digitally voice-recorded and transcribed verbatim for data analysis. Data collection ended once the researcher was hearing the same comments repeatedly from new participants indicating that data saturation was being reached. All information specific to social media was pulled from the interviews to answer the research question. Participant names and identifying information were replaced with pseudonyms.

### Data analysis

We employed inductive thematic analysis whereby themes were drawn inductively from the data. Theoretical concepts have not been imposed on the data, only those that coincide with the data have been used. Analysis went through the steps of familiarisation, initial coding, searching for themes based on initial coding, review of themes, theme definition and labelling (following Braun and Clarke, 2006). Data analysis was conducted primarily by the first author who carried out the analysis by hand. The first author sequentially read through each transcript, writing a face sheet for each participant which summarised contextual information, issues emerging and the author’s reflections. This was followed by making notes about potential themes and key experiences across participants. The first author then re-read each transcript, ensuring that key experiences had not been missed. Initial codes were discussed with the second author and following these discussions codes were grouped into themes and subthemes by the first author. Both authors reviewed these themes, with the first author re-reading the transcripts once more, and the second author reading the transcripts to review the themes. Once a consensus had been reached between both authors through in-depth discussions about the data and proposed themes, final themes were defined and labelled by both authors. Participant quotes have been used to illustrate each theme.

## Findings

Eleven participants described both positive and negative impacts of social media use which required a delicate balancing act to manage, leading to the overarching theme ‘A balancing act’. Four participants (two male, two female) only described positive experiences with social media, but balancing both positive and negative impacts of social media was the overriding experience.

### A balancing act

*Social media comes with good and bad sides, doesn’t it? (‘Emily’)*

*I think there is a bit of a balance there. . .if you live on social media too much it can become very overwhelming. But on the same side there’s a lot of good stuff that goes on. . . (‘Anna’)*

**Figure 1.**
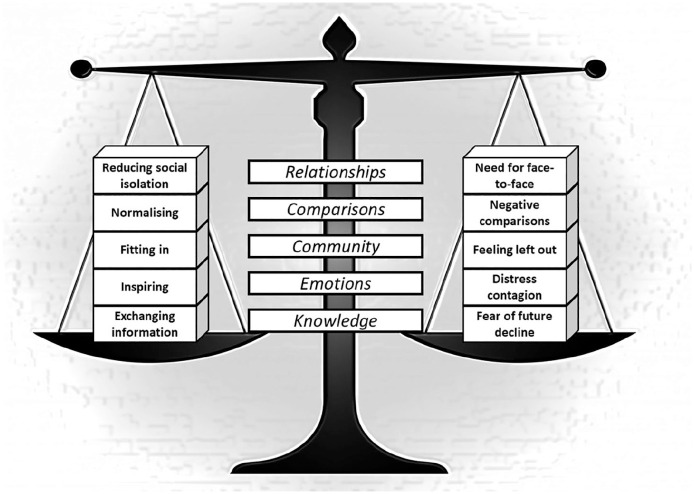
Visual representation of the theme ‘a balancing act’ and related subthemes.

The overarching theme was that social media use came with both ‘good and bad sides’ (i.e. with beneficial and detrimental effects), which required a balancing act to manage. At different times, and under different circumstances, the balance could be tipped to one side (either ‘good’ or ‘bad’) and this required self-reflection and awareness in order to manage when and how to best utilise social media for the purpose of helping to live well with their LTCs. Within this overarching theme we identified the following subthemes: (1) Relationships: reducing social isolation versus need for face-to-face contact; (2) Comparisons: normalising versus negative (upward) comparisons; (3) Community: fitting in versus feeling left out; (4) Emotions: inspiring versus distress contagion; (5) Knowledge: exchanging information versus fear of decline. See Figure 1 for a visual representation of these themes.

### Relationships: Reducing social isolation versus need for face-to-face

Both social media-based communities for people with LTCs, and social media use generally; were described as reducing social isolation in some capacity by eight participants. In terms of online health communities, participants described feeling less alone and isolated as a result of a shared understanding and empathy amongst group members:

. . .it’s reassuring. . .hearing that you’re not the only one suffering silently. Like, if someone says, ‘Oh, I was in class the other day, and I soiled myself, and it was really embarrassing, and everyone was laughing’, it’s terrible, but it’s a way that everyone can-‘Oh yes, I experienced that when I was at work. . .’, it’s nice to know that you’re not the only one. . .there’s so much social media that can help. . .I could imagine, before that happening, I would’ve felt even more isolated. (“James”)

. . .it can be intriguing to see how other people deal with things. . .sometimes they post questions. . . ‘Oh, do you have this symptom or is it just me?’ Sometimes you’re like, ‘Oh, yes. I get that, too’. . .it makes you feel like you’re not alone. . . (‘Ruth’)

One participant also described how one of these groups led to a newly developed friendship for one of her friends who lived with a different LTC:

My friend who has Complex Regional Pain Syndrome. . .when she was finally diagnosed she literally just sat at home and wouldn’t talk to anyone. . .She met a girl on Facebook, in a support group and they are like best friends now. . .It’s so nice because she’s got someone to talk to and I can just see how happy she is. . .having someone to share that with who completely understands. (‘Zoe’)

Three participants talked about how SNSs facilitated them being more open about their condition and their struggles. This was particularly the case for conditions that participants felt had stigma attached to them, but also for many young adults who felt that simply being a young adult with a LTC had stigma attached to it due to societal assumptions that young people are healthy. This meant that they did not feel that they could be as open about their conditions and experiences in everyday life. Therefore SNSs provided a form of anonymity and a safe space to be open and honest about their experiences:

. . .I do find it [*social media group*] helpful. I think a lot of people with Irritable Bowel Syndrome share the same kind of stigma about not wanting to talk about it, because it’s something that people find gross, or it’s not something young people love to talk about. . .luckily, with the internet. . .there’s a lot of support that you can get without having to embarrass yourself or be public about it. . . (‘James’)

I’ve been able to have very frank discussions about Graves’ Disease and the mental health issues that have come with it, with people of my own age, which I don’t think I would have ever had without it. (‘Emily’)

For one participant, sharing about her condition on social media more broadly (i.e. not just in an online health community) led to her re-connecting with old friends who had similar experiences to her in terms of illness, which led to bonding and renewed relationship:

I have been able to connect with some people that have had the same problems. Some of those people have had Graves’ Disease. . .some have had various different things. . .I have connected with a lot of old school friends who I haven’t spoken to in, like, 20 years, over similar sorts of issues. . .and I think that’s helped a lot. . .[*social media*] has made me reconnect with others. . . (‘Emily’)

One participant also reported that SNSs such as Facebook and Instagram helped her to maintain existing friendships and keep up-to-date with her friends’ social activities. The remote nature of this interaction, that could be experienced even when unable to be physically present with friends, was emphasised:

It helps in that when I am in home in bed, I’m still connected to all my friends, so I can still see on social media what people are up to, and I’m not missing out in that way. (‘Rebecca’)

Despite a number of participants citing reduced social isolation and developed or maintained friendships as a result of social media, four participants expressed the desire for face-to-face contact with other young adults, including young adults living with LTCs. This was as opposed to, or in addition to, online contact. It was felt that online interaction could only go so far in reducing social isolation, and that face-to-face contact was the preferred approach.

I think it would be really helpful to try and get these people together locally. To meet people your age that are going through the same thing. . .with social media, you can keep in touch with those people. But if they’re local as well, you can meet up with them. I think that would be really, really helpful, just bringing those people together face-to-face to actually discuss these sorts of things. (‘Ella’)

### Comparisons: Normalising versus negative comparisons

Social media-based communities for people with LTCs were described by three participants as helping to normalise their experiences through hearing about, and comparing themselves to others who have had similar experiences, for example:

. . .it makes you realise how kind of normal you are in the grand picture because you think that you’re really abnormal. . . (‘Samantha’)

The above quote from ‘Samantha’ highlights how a Facebook group for people living with lupus helped normalise her experiences. Her life with lupus fell outside of broader social norms (making her feel ‘abnormal’), but within this online community she became ‘normal’ as she identified similarities between herself and others in the group. However, social media use more broadly could lead to participants making negative (‘upward’) social comparisons to healthy peers perceived to be ‘superior’, which *exacerbated* feelings of abnormality and inadequacy by seeing the fun things that others are doing/how others look. These factors then highlighted their inability to fit into broader social norms (e.g. be able to do certain things or look certain ways) which negatively impacted on their mental wellbeing and self-worth, as described by two participants:

. . .you do see people going off and doing amazing, wonderful things that you can’t, and that can be quite difficult to understand or accept. . .I don’t really do social media a lot. . .particularly when I was suffering with depression, logging onto Facebook and seeing everyone out doing wonderful things. . .makes you feel a lot worse really. (‘Emily’)

. . .social media makes you feel like you should have a particular life, and if your life isn’t like that because of your condition, then it’s definitely a negative thing. . .you see all over social media people being really built up, but it wouldn’t be possible for me to ever achieve that kind of body because I wouldn’t be able to go to the gym. . .and I can’t eat enough to be able to sustain a body like that. . .definitely the social pressures of Instagram. . .which then makes you think about how you look. And then when you’ve got something else that’s affecting how you look which you can’t control, then it’s a bit complicated. . .It’s really difficult if you don’t fit the cookie cutter shape, and way of life. If you don’t earn a certain amount to be able to live that kind of life, and if you don’t have that kind of body. . . And then when you’ve got a condition, that only enhances all those things. . . (‘Rebecca’)

### Community: Fitting in versus feeling left out

Five participants described how being part of an online group for people living with the same LTC helped them to fit it and led to a sense of belonging and shared identity, which relates to the shared understanding and normalising that occurred within the groups:

. . .before [having lupus]. . .I didn’t really have an identity. . .But now I kind of see myself as a lupus mummy and there’s lots of kind of different support groups, on Facebook mainly, where it makes you realise how kind of normal you are in the grand picture. . .and. . .people accept you. (‘Samantha’)

However, four participants described the theoretical benefits of online communities for helping to live well with their LTCs, but expressed frustration at not being able to find ones which suited their unique needs/situations. Some found that online communities predominantly comprised older people, people with a greater severity of the condition, or people predominantly of the opposite gender (in the case of one male participant); and they therefore felt unable to relate to others in the group. Others reported that due to having multiple conditions they struggled to find the best-suited group for their multiple needs and felt that they did not fit in with the groups available.

. . .I often think, ‘Should I be part of that group?’ With say chronic fatigue. . .it’s peaks and troughs and I would kind of feel, ‘Should I be there? Am I bad enough to be there?’ And the same with my thyroid, typically people that have had their thyroid removed are probably 30 years older than me. . .there are not that many people out there my age that have gone through what I’ve gone through. . .so, it may not be entirely relevant to me. . . (‘Ella’)

. . .you feel a bit like you’re watching from the outside, and you’re like, oh yes, I connect with that, and that. . .but nothing specific. . .because you don’t fit into these categories. . .Because if you were just able to chat with other young people who are in the same boat, you’d be able to laugh about it a bit more, I think, rather than feel quite alone because of, you don’t fit. Because you’re already ostracised because of your condition, and then if you don’t fit a box in that condition, then you don’t have anywhere to go to, to talk to anyone about it. (‘Rebecca’)

### Emotions: Inspiring versus distress contagion

Three participants reported that reading things shared on social media by others living well with their LTCs was inspiring, uplifting and encouraging:

. . .there’s a Crohn and Colitis charity. . .I have signed up to their Facebook page. It is quite encouraging to see the stories that they post of people who are living really good lives and of the research that they’re doing. . .that’s encouraging. (‘Anna’)

. . .on Facebook [you] get all these kind of inspiring memes about life with lupus. . .it puts a smile on your face. . .it does help. . . (‘Samantha’)

It’s like a post on Tumblr I used to see, the woman said, ‘Three years ago I was lying on the bathroom floor, crying, and now today I’ve just bought my first house with my husband. . .’ It’s empowering to know that people can get through this. . . (‘Alice’)

However, four participants reported that the sharing of negative experiences on online health communities could have negative emotional impacts on them:

It was so depressing. Everybody was, ‘Oh, I’m having a really bad day today.’ I just thought, ‘This is going to drag me down.’ So, I stopped going on there. (‘Elizabeth’)

It can be a positive or it can be a negative because you do get people who wallow. . . (‘Tom’)

I would say that I don’t go on it that much, because I tend to find there’s a lot more depressing stories on there, there is a lot of moaning. (‘Anna’)

### Knowledge: Exchanging information versus fear of decline

Six participants referenced sharing and gaining of knowledge related to their LTC/s through social media. With regard to online health communities, participants talked about the mutual sharing of experiential knowledge. For example, regarding experiences of medications/treatments, coping strategies, and eating well:

They give you ideas of things that you can do at home with your children if you’re not feeling well enough to go outside, so that your kids aren’t just kind of stuck in front of the telly while you have a nap. There’s recipes as well, because I find that cutting out a lot of the processed food really helps my symptoms. . . (‘Samantha’)

It’s interesting to see how other people’s reactions are to the drug and how people’s reactions are to Multiple Sclerosis full stop. . .I go on the board quite a lot when I’m having a relapse because it’s seeing how other people have adapted to that. . .I find those message boards good and quite informative. . . (‘Tom’)

However, reading about others whose condition had deteriorated further than their own, led to fearing their own future decline. In these cases, gaining information and knowledge was not always helpful:

It can be quite scary as well, seeing how far the condition can go and what potentially might happen to me. I think I would rather not know too much and not over analyse too much. Again, I think there is a bit of a balance there, I think if you live on social media too much it can become very overwhelming. But on the same side there’s a lot of good stuff that goes on and it’s nice to know that there are people researching to find cures and to find ways of helping people live better lives. . . (‘Anna’)

As the above quote from ‘Anna’ also highlights, social media was also seen as providing an opportunity for sharing new research about LTCs. This was also highlighted by two other participants, for example:

I found out about the hypobaric chamber treatment through Facebook. . .My cousin posted an article saying, ‘Fibromyalgia can be cured by hypobaric oxygen chamber’. . .the study said that 70% of the people involved received significant or total improvement. . .I just thought, ‘Wow. That’s got to be worth a try.’ (‘Elizabeth’)

Two participants also described finding memes (images that aim to convey a particular phenomenon, theme, or meaning) on Facebook pages for people with their condition, and then sharing it on their own Facebook timeline to be seen by their friends. This was seen as a way of raising awareness of the condition and its impacts amongst their wider friends:

See, I ‘liked’ a group on Facebook to do with chronic fatigue. . .those pictures with the inspirational quotes. . .I did lift one this time, saying something like, ‘I’m sorry if I’m not able to respond to your text. Sorry I’m not able to-’ just so that people actually- You know, my friends do say, ‘Okay, she genuinely can’t do it.’ (‘Ruth’)

Pages that I’ve ‘Liked’ on Facebook, they post little images and they’ll have a little slogan. . .At the time, I was really struggling to make other people understand what I was going through and they’d just summarise it perfectly. So, it would be, ‘I am not being lazy, I am ill. I am struggling for x, y and z. . .’ That, kind of helped, because I’d ‘Like’ it and then hope that anybody who was fond of me. . .would then go, ‘Oh, okay.’ I did save a few and show them to my partner. Some of them made him quite upset, he’s like, ‘Really? That’s what you go through?’ (‘Elizabeth’)

Raising awareness of LTCs was also mentioned by ‘Luke’ as an important role played by social media:

I think awareness is a really good thing. . .that people do understand that it’s a real challenge for the people experiencing it. . .I think [*social media*] is very important for helping people understand. (‘Luke’)

## Discussion

The aims of this paper were to explore the role social media (including online health communities) plays in helping and/or hindering young adults to live well with their LTCs. The overarching theme was ‘a balancing act’. For most participants, social media use came with both ‘good and bad sides’, which required a balancing act to manage. At different times the balance could be tipped to one side and this required self-reflection and awareness in order to manage when and how to best utilise social media for the purpose of helping them to live well with their LTCs. The following subthemes were explored: (1) Relationships: reducing social isolation versus need for face-to-face; (2) Comparisons: normalising versus negative (upward) comparisons; (3) Community: fitting in versus feeling left out; (4) Emotions: inspiring versus distress contagion; and (5) Knowledge: exchanging information versus fear of decline. The uniqueness of this paper is that it explores social media use for young adults specifically, and explores not only the use of online health communities, but also the broader use of social media. Some of the findings align with existing research with older populations; however, some of the previously reported benefits were not reported by this exclusively young adult sample, and additional positive impacts were reported.

Both social media-based communities for people with LTCs, and social media use generally; were described as reducing social isolation. Feeling less alone and isolated, having increased social support, experiencing shared understanding and empathy, and engaging in reciprocally supportive relationships; were some of the benefits described by participants which align with earlier research (e.g. Allen et al., 2016; Kingod et al., 2017; Patel et al., 2015). Additional positive social impacts were reported. SNSs were seen as providing a means to discuss more openly about their condition and their struggles (more so than in ‘real life’) which was seen as particularly valuable in light of the perceived stigma attached to having a LTC in young adulthood. This stigma sometimes restricted such openness in wider friendship circles and social media paved the way for safe sharing of experiences without fear of stigma or judgement. This aligns with the literature on social media-based behaviour change interventions, which highlights that the potential anonymity of online groups allows participants to feel empowered by being in a safe environment, which is particularly beneficial in cases where health topics may be considered ‘taboo’ (Santarossa et al., 2018). Another novel finding from the current study was that SNSs helped young adults with LTCs to maintain existing friendships as they were still able to see what social activities were happening, stay up-to-date with the lives of friends, and communicate with friends without relying on face-to-face contact. The remote capacity of this interaction meant that this social inclusion was not dependent on wellness; and although face-to-face contact was preferred, staying connected via social media was seen as better than no contact. However, it was still emphasised that online interaction could only go so far in reducing social isolation, and that social media did not provide a replacement for the need for face-to-face contact, building on earlier work (Attard and Coulson, 2012; Caron and Light, 2015).

Social media-based communities for people living with LTCs were also seen to help normalise individual’s experiences through comparisons to similar others. This was in direct contrast to wider use of social media which could reinforce feelings of abnormality by highlighting how individual’s lives differed from social norms amongst their age group. Wider use of social media led to making negative (upward) social comparisons to peers, exacerbating feelings of inadequacy and abnormality by seeing the fun things that others were doing and how they looked. This then highlighted participants’ inability to do certain things or look certain ways which negatively impacted on their mental wellbeing and self-worth. This follows on from research with healthy adolescents and young adults, whereby social comparisons through social media have been found to lead to lower self-esteem, lower wellbeing, and body image dissatisfaction (e.g. Gothenburg Research Institute, 2012; Santarossa and Woodruff, 2017). We have found that this may also be the case for some young adults with LTCs. Negative social comparison is thought to motivate the individual to engage in self-enhancement and self-improvement in order to meet the comparison standard (e.g. Higgins, 1987); however, in the case of young adults with LTCs the condition may prevent them from being able to achieve this standard (e.g. through being unable to go out socialising, go travelling, earn more money, or achieve a particular body shape). Therefore, social comparison is potentially more harmful for young adults with LTCs as it could merely facilitate the identification of the discrepancy for the mismatch between the self and peers and the motivation to ‘improve’ the self; potentially without the ability to do so.

Participants described how being part of an online health community facilitated a sense of belonging, with their descriptions aligning with Yuval-Davis’ (2004, 2011) definition of belonging as a feeling that one fits in, belongs and is in a safe space. Participants also described the formation of a shared identity within online health communities, building on Conrad et al.’s (2016) postulation that these communities lead to collective illness identities. These are pertinent findings as belonging has been described as a key determinant of health and wellbeing (e.g. Bernat and Resnick, 2009; Yuval-Davis, 2004). However, a unique finding from the current study was that others felt that they *didn’t* fit in to the social norms of online health communities due to issues around age (with the groups predominantly comprising older adults), gender (in the case of one male participant), condition severity, and living with multiple conditions. Therefore, not only can young adults feel left out from the interactions and experiences posted by their peers on social media, they can also feel left out from social media groups set up specifically for individuals living with LTCs.

Participants also reported that reading about others living well with LTCs on online communities could be inspiring, encouraging and uplifting; however, reading about negative experiences could be distressing. This supports the descriptions in the literature of distress contagion from participating in online health communities (e.g. Fergie et al., 2015, 2016; Kohut et al., 2017; van Uden-Kraan et al., 2008). The sharing and gaining of knowledge related to LTC/s through social media was also described by participants. With regard to online communities, participants talked about the mutual sharing of experiential knowledge related to treatments/medications and coping strategies. This expands on Fergie et al.’s (2015, 2016) research, who described the useful sharing of research through social media and experiential information through social media-based groups for people with diabetes or mental ill health. However, as with the above descriptions of distress contagion, this sharing could have negative impacts. For example, sharing about declining health could lead to fear of their own future decline.

Social media was also seen as providing an opportunity for raising awareness about LTCs amongst friends and family, through sharing of memes which portrayed an accessible message about the condition, and research. This follows on from Gonzalez-Polledo’s (2016) research with Tumblr users who experienced chronic pain and illness, who described the sharing of memes as a way to raise awareness and to communicate their illness. The need for raising awareness and reducing stigma about LTCs has been identified in previous research (e.g. Lambert and Keogh, 2015) and social media may offer a way of facilitating this.

Participants described both the ‘good and bad’ of social media, effectively weighing up the risks and benefits of its use and developing their own strategy for its use that maximises the benefits and minimises the harmful effects. This may shed some light on some contradictory findings reported in the wider literature. For example, in a systematic narrative review of social media use for adolescents; social media was reported to: increase social support, but also increase social isolation; to increase self-esteem, reduce social anxiety and promote mental health, but also increase risk of depression (Best et al., 2014). The current research provides some insight into these supposed contradictions. For individuals with LTCs, social media can be both a help and a hindrance to living well with their LTC/s and it may be helpful to identify for themselves which aspects of engaging with social media are helpful and which are not. For example, if an individual notices that regularly going on Facebook and seeing other people travelling, socialising, and doing things that the individual is currently upset about being unable to do; it may be worth limiting time spent on Facebook and spending that time doing something that serves as a distraction/uplifts their mood. Likewise, if an individual is noticing that when they are feeling well they find it unhelpful to read negative posts on social media-based groups for their LTC as it worsens their mood and makes them fearful for the future; then it is perhaps worth not going on those groups when feeling well. But on the other hand, if when they are unwell those groups help them to feel less alone and provide an outlet to express negative feelings to others who understand, it will be beneficial to utilise it at those times. Self-awareness of the impact of social media on mood/wellbeing appears to be key in best utilising social media when living with a LTC in young adulthood.

It is important to acknowledge some limitations of this research. The current sample may not be fully representative of the wider population of young adults with LTCs due to the nature of those who self-selected to take part. The sample were predominantly female; all able to work or study in some capacity; and all were from a white ethnic background. This should be taken into account when considering the findings.

In summary, this study adds to the limited research exploring social media use amongst young adults with LTCs. Findings revealed that social media use comes with both ‘good and bad sides’, which requires a delicate balancing act to manage. At different times the balance could be tipped to one side (either ‘good’ or ‘bad’) and this required self-reflection and self-awareness in order to manage when and how to best utilise social media for the purpose of helping to live well with their LTCs. Social media was reported to reduce social isolation, but did not replace the need for face-to-face contact; making comparisons to others through social media could be normalising or could highlight perceived inferiority to ‘superior’ others; social media helped individuals to feel that they fit in and belong, but could also leave them feeling left out; posts on online communities could be inspiring and encouraging, or cause distress; and social media facilitated the sharing and gaining of new knowledge, but not all knowledge was positive and could lead to fear of decline. Individuals identified both the risks and benefits of using social media when living with a LTC in young adulthood; going through a process of working out how best to utilise social media for their own needs, including identifying when it is a hindrance to them living well. It is recommended that young adults with LTCs become self-aware about the impact of their social media use on their wellbeing, and identifying times when limited use of social media may be preferable. Being aware of making negative (upward) social comparisons to others according to how they present themselves on social media, is especially important for young adults with LTCs. Young adults with LTCs have heightened awareness of their discrepancies with societal expectations of young adulthood which they may not be able to attain due to their condition. Likewise, whilst there is indeed a ‘good’ side to social media use for young adults with LTCs, and future self-management research should continue to explore the role of social media; this should not be the sole focus as participants also desire face-to-face contact with peers in order to effectively reduce their feelings of social isolation.
